# Senecavirus A 3C Protease Mediates Host Cell Apoptosis Late in Infection

**DOI:** 10.3389/fimmu.2019.00363

**Published:** 2019-03-13

**Authors:** Maureen H. V. Fernandes, Mayara F. Maggioli, Jaelin Otta, Lok R. Joshi, Steve Lawson, Diego G. Diel

**Affiliations:** Animal Disease Research And Diagnostic Laboratory, Department of Veterinary and Biomedical Sciences, South Dakota State University, Brookings, SD, United States

**Keywords:** Senecavirus A, Seneca Valley virus, apoptosis, 3C protease, virus egress

## Abstract

Senecavirus A (SVA), an oncolytic picornavirus used for cancer treatment in humans, has recently emerged as a vesicular disease (VD)-causing agent in swine worldwide. Notably, SVA-induced VD is indistinguishable from foot-and-mouth disease (FMD) and other high-consequence VDs of pigs. Here we investigated the role of apoptosis on infection and replication of SVA. Given the critical role of the nuclear factor-kappa B (NF-κB) signaling pathway on modulation of cell death, we first assessed activation of NF-κB during SVA infection. Results here show that while early during infection SVA induces activation of NF-κB, as evidenced by nuclear translocation of NF-κB-p65 and NF-κB-mediated transcription, late in infection a cleaved product corresponding to the C-terminus of NF-κB-p65 is detected in infected cells, resulting in lower NF-κB transcriptional activity. Additionally, we assessed the potential role of SVA 3C protease (3C^pro^) in SVA-induced host-cell apoptosis and cleavage of NF-κB-p65. Transient expression of SVA 3C^pro^ was associated with cleavage of NF-κB-p65 and Poly (ADP-ribose) polymerase (PARP), suggesting its involvement in virus-induced apoptosis. Most importantly, we showed that while cleavage of NF-κB-p65 is secondary to caspase activation, the proteolytic activity of SVA 3C^pro^ is essential for induction of apoptosis. Experiments using the pan-caspase inhibitor Z-VAD-FMK confirmed the relevance of late apoptosis for SVA infection, indicating that SVA induces apoptosis, presumably, as a mechanism to facilitate virus release and/or spread from infected cells. Together, these results suggest an important role of apoptosis for SVA infection biology.

## Introduction

Senecavirus A (SVA) is a non-enveloped single-stranded positive-sense RNA virus of the genus *Senecavirus*, family *Picornaviridae* (1, 2). SVA was first detected as a cell culture contaminant in 2002 in the United States (US) (3), and subsequently identified as a novel picornavirus closely related to members of the genus *Cardiovirus* (1). The SVA genome is approximately 7.2 kb in length containing a single open reading frame (ORF) that encodes a 2181 aa polyprotein, which is cleaved into four structural proteins (VP1, VP2, VP3, and VP4) and eight non-structural proteins (L, 2A, 2B, 2C, 3A, 3B, 3C, and 3D) (1). Processing of the polyprotein into mature viral proteins is catalyzed by the non-structural protein 3C^pro^, a virus-encoded cysteine protease that contains a conserved His, Asp, Cys catalytic triad (1, 4). While the structural proteins of picornaviruses form the virus capsid and are involved in receptor binding and cell entry, non-structural proteins are mainly responsible for virus replication (5) and play important roles on virus-host interactions contributing to innate immune evasion, virus virulence and pathogenesis (6–28).

Since its identification, SVA has been associated with sporadic cases of vesicular disease in pigs in the US and Canada (29–31). However, after 2014, outbreaks of vesicular disease associated to SVA have been reported in major swine producing countries around the world (32–36). The lesions observed during these outbreaks include vesicles on the snout, oral mucosa and feet, involving the coronary bands, interdigital space, due claws, and/or sole (29, 31, 33, 34, 37, 38). This clinical presentation was also observed in experimentally infected animals (39–42). Importantly, SVA-induced disease is clinically indistinguishable from other high consequence vesicular diseases of swine, including foot-and-mouth-disease (FMD), swine vesicular disease (SVD), vesicular stomatitis (VS), and vesicular exanthema of swine (VES) (31, 43).

In addition to its relevance to animal health, SVA has been tested as an oncolytic agent for cancer treatment in humans (2, 44–47). Given the promising results in animal models, SVA was tested in phase I clinical trials, becoming the first oncolytic picornavirus to be tested in humans (47, 48). The main limitations to the broad use of SVA as an oncolytic agent in humans, however, are the development of neutralizing antibodies that result in rapid viral clearance from treated patients and the fact that the molecular basis of SVA's oncolytic activity remain unknown (49). A better understanding of the molecular SVA-host interactions and of the mechanism(s) underlying virus replication in susceptible cells may allow the development of improved SVA-based therapeutics for cancer treatment.

Picornaviruses modulate many host cellular pathways, including the host translation machinery, innate immune responses and cell survival or apoptosis. Foot-and-Mouth disease virus (FMDV), for example has been shown to inhibit nuclear factor kappa B- (NF-κB) (18) and interferon beta (IFN-β) signaling (28). Enteroviruses, on the other hand, were shown to take advantage of the host secretory autophagy pathway to enhance their transmissibility (50) and cardioviruses were shown to inhibit nucleocytoplasmic trafficking of host cell proteins (7). Another important cellular process that is targeted by several picornaviruses is programmed cell death, or apoptosis. Poliovirus has been shown to modulate apoptosis and is known to inhibit or induce host cell death during different phases of the infection (51, 52), while Coxsackievirus B3 (53), and Hepatitis A virus (54) are known to induce apoptosis. Recently, apoptosis was observed in lesions caused by FMDV in the tongue of experimentally infected pigs (55). These observations highlight the importance of modulation of host cell apoptosis for the infection biology of picornaviruses.

While apoptosis usually functions as a host defense mechanism that ensures killing of infected cells (56, 57), several viruses, including picornaviruses, have been shown to induce apoptosis to enable efficient virus transmission while avoiding overt inflammatory responses and activation of the immune system (58). Activation of apoptosis occurs mainly by two distinct pathways, the intrinsic and extrinsic pathways, which utilize executioner caspases (Casp–3, –6, and –7) to induce cell death (56, 59). Caspases are a family of serine proteases that mediate many features of apoptosis (57). These enzymes are maintained in the cell cytoplasm as inactive proenzymes comprising two subunits (large and small) and a variable amino terminal prodomain. Activation of the caspases requires loss of the prodomain through catalytic cleavage of a C-terminal aspartate residue and dimerization of the large and small subunits to form the active protease (57, 60). Apoptotic responses are usually initiated by activation of Casp-8 or Casp-9 (via tumor necrosis factor receptor or Fas, respectively), whose activity results in downstream activation of the effector Casp–3, –6, and –7 (57, 60). These effector enzymes cause cellular disassembly through cleavage of cell death substrates, including lamin, poly(ADP-ribose) polymerase (PARP) or the caspase-activated DNase/DNA fragmentation factor (CAD/DFF) complex (61–63). Additional substrates of the effector caspases include pro-survival transcription factors, such as NF-κB (64, 65). Collectively, the action of the caspases result in fragmentation of cellular DNA and cell death (57, 62, 63).

NF-κB is a ubiquitous transcription factor that modulates not only cell death but also innate immunity and inflammatory responses. This pathway seems to play critical roles in the picornaviral life cycle (8, 11, 14, 16, 18, 19, 22, 27, 66). Activation of the NF-κB signaling pathway by viruses is mediated by pattern recognition receptors (PRRs) which detect pathogen associated molecular patterns (PAMPs; including double-stranded RNA [dsRNA] and/or viral proteins) and initiate the signaling cascade that leads to host gene transcription (67, 68). In unstimulated cells, the NF-κB transcription factors (NF-κB-p50, -p52, -p65, RelB, or c-Rel) form homo- or heterodimers (69), that are sequestered by the NF-κB inhibitor alpha (NFκBIA/IκBα) in the cell cytoplasm (70). Once the pathway is activated, IκBα is phosphorylated by upstream IκB kinases (IKKα, IKKβ), triggering its proteasomal degradation and leading to translocation of NF-κB subunits to the nucleus, where they undergo additional post-translational modifications and modulate transcription of pro-inflammatory-, innate immunity-, and/or apoptosis-related genes (69). In this context, NF-κB seems to play an essential role in protecting host cells from picornavirus-induced apoptosis (71).

In the present study, we investigated the host apoptotic responses during SVA infection and dissected the interplay between the virus, host cell apoptosis and NF-κB signaling. Results here show that SVA induces apoptosis late in infection, which plays a critical role on the virus infection cycle, likely facilitating virus release from infected cells.

## Materials and Methods

### Cells and Virus

Primary swine turbinate (STu) and NCI-H1299 non-small human lung carcinoma cell lines (ATCC® CRL-5803) were cultured at 37°C with 5% CO_2_ in minimum essential medium (MEM, Corning®) or RPMI 1640 medium (Corning®) supplemented with 20 or 10% fetal bovine serum (FBS; Seradigm), respectively. Cell culture media was supplemented with 2 mM L-glutamine (Corning®), penicillin (100 IU/mL; Corning®), streptomycin (100 μg/mL; Corning®), and gentamicin (50 μg/ml; Corning®). Senecavirus A strain SD15-26 was isolated from a vesicular lesion from a finishing pig and has been previously characterized (39). Low passage (passage 4) SVA stocks were prepared and titrated in H1299 cells, and used in all experiments involving SVA infection described here.

### Plasmids

Luciferase reporter plasmids (pNF-κB-luciferase reporter, and pRL-TK control plasmids) are commercially available (Promega). The coding sequence of SVA 3C^pro^ was fused with Flag-tag (N-terminus fusion), chemically synthesized (GenScript), and cloned into the pcDNA3.1 expression plasmid (pcDNA-Flag-3C). Additionally, SVA 3C^pro^ was amplified from SVA strain SD15-26 by RT-PCR and cloned into the eukaryotic expression plasmid pET28a as a His-tag fusion protein (pET28-SVA-3C). SVA strain SD15-26 VP1 expressing plasmid (pcDNA-HA-VP1) was kindly provided by Dr. Steve Lawson (Department of Veterinary and Biomedical Sciences, South Dakota State University). Control plasmid pCMV-Flag-BAP is commercially available (Sigma). The plasmid pCMV-HA-NF-κB-p65 was constructed by standard cloning techniques. Briefly, porcine NF-κB-p65 coding sequence (GenBank NM_001114281) was PCR amplified from cDNA prepared from swine testicle (ST) cells and cloned in fusion with the HA epitope tag into the pCMV-HA-N expression vector (Clontech). Sequence identity of the expression plasmids generated here were confirmed by DNA sequencing.

### Antibodies and Reagents

Antibodies against β-actin (clone C4), NF-κB-p65 (clone C-20; C-terminus), NF-κB-p65 (clone F-6), and IκBα (clone H4) were purchased from Santa Cruz Biotechnology. Antibodies against NF-κB-p65 (clone C22B4; N-terminus), IKKα (clone3G12), IKKβ (D30C6), cleaved caspase-3 (Asp175, clone 5A1E), and PARP (clone 46D11) were obtained from Cell Signaling. SVA VP1 and SVA VP2 mouse monoclonals and SVA whole virus antibodies were kindly provided by Dr. Steve Lawson (SDSU). Anti-Flag or anti-HA mouse monoclonal antibodies are commercially available (GenScript and Thermo Scientific, respectively). Anti-rabbit and/or anti-mouse secondary antibodies conjugated with Alexa Fluor® 594 and Alexa Fluor 488® were purchased from Life Technologies. IRDye 800CW-labeled anti mouse IgG and IRDye 680-labeledRD anti rabbit IgG secondary antibodies were purchased from Li-Cor Biosciences. Recombinant TNF-α was obtained from InvivoGen. Staurosporine was purchased from Cell Signaling and Z-VAD-FMK was obtained from Santa Cruz Biotechnology.

### SVA 3C^pro^-Specific Rabbit Antibody Production

SVA 3C^pro^ was expressed using a prokaryotic expression system. SVA 3C^pro^ coding sequence (GenBank KX778101) was PCR amplified from cDNA of SVA SD15-26 and cloned into the bacterial expression plasmid pET-28a (EMD Millipore/Novagen). The recombinant protein was expressed in *Escherichia coli* strain BL-21 cells as a 6×His-tag fusion protein and purified using nickel-charged agarose resin (Qiagen) according to the manufacturer's instructions. Purified recombinant protein was used for polyclonal antibody production in rabbits.

One adult 12-week old rabbit was kept in an individual cage with food and water *ad libitum* throughout the study. The animal was immunized with 660 ng of SVA 3C^pro^ emulsified in water-in-oil (W/O) adjuvant (1:1; 1 mL:1 mL; Montanide™ ISA 50 V2, Seppic). The antigen was administered by three 0.5 mL subcutaneous injections and one 0.5 mL intramuscular injection. Two weeks post-primary immunization the animal received a booster immunization as above. Two weeks after the booster immunization the rabbit was euthanized and exsanguinated, and polyclonal serum containing SVA 3C^pro^-specific antibodies was isolated by centrifugation (at 2,000 × g for 15 min at 4°C). All animal procedures and protocols for antibody production were reviewed and approved by the South Dakota State University Institutional Animal Care and Use Committee (IACUC) under approval number 15-095A.

### Terminal Deoxynucleotidyltransferase-Mediated dUTP-Biotin Nick-End Labeling (TUNEL)

Primary STu cells were cultured on glass cover slips and infected with SVA (MOI = 5) or mock-infected and then fixed at 12 h p.i. with 3.7% formaldehyde in PBS (pH 7.2) for 20 min. Positive control cells were treated with 0.1 μM staurosporine for 4 h and then fixed as above. Additionally, formaline-fixed paraffin-embedded (FFPE) tissue sections from a previous SVA pathogenesis experiment conducted in our laboratory (40) were subjected to TUNEL. Cell cultures or tissue samples were permeabilized and stained with an *in situ* cell death detection kit (TUNEL, Abcam) according to the manufacturer's instructions.

### Histopathology

Formalin-fixed paraffin-embedded tissue sections (skin) from a previous SVA animal experiment were processed following standard histological procedures and stained with hematoxylin and eosin for histological examination. All animal procedures and protocols were reviewed and approved by the SDSU IACUC under approval number 16-002A.

### *In situ* Hybridization

Formalin-fixed paraffin-embedded tissue sections (skin) were used to perform *in situ* hybridization (RNAScope®). ISH probe utilized was specific for the viral genome SVV (301-1345 region of VP1 gene, GenBank: EU271758.1, Advanced Cell Diagnostics, Inc.). The ISH was performed as previous described (72).

### Flow Cytometry

Activation of caspase 3 and 7 (Casp-3/-7) was assessed by flow cytometry during SVA infection. Semi-confluent STu or H1299 cells were cultured in 12-well plates and infected with SVA (MOI = 5). At 2, 4, 5, 6, 7, 8, and 10 h p.i. the supernatant was removed and the cells trypsinized to dissociate the monolayer. Mock-infected cells and mock-infected cells treated with 0.3 μM staurosporine were used as negative and positive controls, respectively. After trypsinization, cells and supernatant were mixed and centrifuged at 1,500 × g for 5 min at 4°C. Activation of Casp-3/-7 was assessed using the CellEvent™ Caspase-3/7 Green Flow Cytometry Assay kit (Thermo Fisher Scientific) according to manufacturer's protocol. The flow cytometry data were acquired with an Attune NxT flow cytometer (Thermo Fisher Scientific) and analyzed using FlowJo software (TreeStar).

To investigate activation of Casp-3/-7 in the context of NF-κB-p65 expression and SVA infection, STu cells were transfected with 1 μg of pCMV-HA-NF-κB-p65 or empty pCMV-HA plasmids (control) using Lipofectamine 3000 (Life Technologies) and subsequently infected with SVA (MOI = 5). Cells were collected at 4, 7, 8, and 10 h p.i. and processed for flow cytometry assessment of Casp-3/-7 activation as described above.

### Growth Curve

SVA growth curves were performed in STu and H1299 cells. Cells were cultured in 12-well plates, inoculated with SVA (MOI = 5) and harvested at 2, 4, 8, 12, and 24 h p.i. Virus titers were determined on each time by limiting dilutions in H1299 cells. At 48 h p.i. cells were fixed (3.7% formaldehyde), permeabilized (0.2% Triton-X), and stained with an anti-SVA whole virus rabbit polyclonal antibody. Viral titers were determined by the Spearman and Karber's method (73) and expressed as tissue culture infectious dose 50 (TCID_50_) per milliliter.

The effect of NF-κB pathway activation during SVA replication was assessed by overexpression of NF-κB-p65 in STu cells. For this, semi-confluent STu cells were plated in 12-well plates and transfected with 1 μg of pCMV-HA-NF-κB-p65 or empty pCMV-HA (control) per well using Lipofectamine 3000 (Life Technologies) as recommended by the manufacturer. At 24 h post-transfection, cells were infected with SVA (MOI = 0.1). After 1 h adsorption, the inoculum was removed and fresh media was added. The cells were harvested at 2, 4, 8, 12, and 24 h p.i. and virus titers determined as described above.

To assess the role of apoptosis on SVA infection cycle, multiple-step growth curves were performed in STu or H1299 cells. Cells were plated in 12-well plates and infected with SVA (MOI = 0.1). After 1 h adsorption, fresh media containing 30 μg/mL of Z-VAD-FMK, a pan-caspase inhibitor was added to the cells. Plain media was added to untreated control wells. Cells and supernatant were collected separately at 2, 4, 8, 12, and 24 h p.i. and subjected to virus titration. Additionally, the effect of apoptosis on SVA infection was assessed in STu cells infected with a high MOI. For this, cells were infected with SVA at an MOI = 5 and cells and supernatant collected separately at 8 h p.i. and subjected to virus titrations.

### RNA Extraction and Quantitative Reverse-Transcription-PCR (RT-qPCR)

To determine transcription levels of NF-κB target genes during SVA infection we performed RT-qPCR. STu cells cultured in 6-well plates were infected with SVA (MOI = 5), collected at 2, 4, 8, and 12 h p.i., and cellular RNA extracted using TRIzol reagent (Invitrogen) according to manufacturer's protocol. RNA samples were treated with DNase (Ambion) and further cleaned using the RNeasy® Mini kit (QIAGEN). Mock infected cells were used as a negative control. Transcription levels of TNF-α, Caspase 8, FADD, BAX, BAK, BCL-2, XIAP, CXCL8, PTGS2, IRF1, and NF-κBIA genes were determined using RT-qPCR and TaqMan gene expression assays (Thermo Scientific). The housekeeping gene glyceraldehyde 3-phosphate dehydrogenase (GAPDH) was used as loading control. RT-qPCR reactions were performed using the RNA-to-Ct™ 1 Step-kit and TaqMan assays for each target gene. The amplification/detection reactions were performed in a 7500 Real Time PCR System (Applied Biosystems). Transcription of target gene was normalized to that of GAPDH and genome copy numbers were determined using the relative quantitation method. Data were analyzed and expressed as fold changes normalized to levels of RNA detected in mock infected cells.

### Indirect Immunofluorescence (IFA)

Expression and activation of NF-κB-p65 was assessed during early stages of SVA infection by IFA. STu cells cultured in 24-wells plates were infected with SVA (MOI = 5). At 0, 2, and 4 h p.i. cells were fixed using 3.7% formaldehyde in PBS (pH 7.2) for 20 min. The cells were washed three times with PBS and permeabilized with 0.2% Triton X-100 in PBS for 10 min at room temperature (RT). Plates were incubated for 1 h at RT with an antibody specific for the C-terminus-NF-κB-p65 (1:250 in PBS/1% BSA) and for SVA (1:250 in PBS/1% BSA). After primary antibody incubation, cells were washed as above and incubated for 1 h at RT with appropriate secondary antibodies conjugated with Alexa Fluor 594 and/or 488 (1:250 in PBS/1% BSA). Cells were washed three times with PBS and nuclear stain was performed with DAPI (Thermo Scientific). Cells were visualized using a fluorescence microscope (Olympus CKX53, 40× magnification).

Expression and activation of NF-κB-p65 was also assessed during late stages of SVA infection by IFA. STu cells cultured in 24-well plates were infected with SVA (MOI = 1). At 7 and 8 h p.i. cells were fixed and IFA was performed as described above.

The effect of SVA 3C^pro^ on expression of NF-κB-p65 was assessed by IFA. H1299 cells were transfected with pcDNA-Flag-3C (100 μg) expression plasmid and subjected to IFA staining. C-terminus-NF-κB-p65 (1:250 in PBS/1% BSA) and Flag-Tag-specific antibodies (1:250 in PBS/1% BSA) were used as primary antibodies. All IFA steps were performed as described above.

### Western Blots

The effect of SVA on apoptosis and NF-κB signaling pathway was investigated by western blots. Semi-confluent monolayers of STu cells cultured in 6-well plates were infected with SVA (MOI = 10) and harvested at 0.5, 1, 2, 3, 4, 8, and 12 h p.i. Mock-infected cells were used as negative controls. This experiment was also performed in H1299 cells, with samples collected at 4, 8, 12, and 24 h p.i. Mock-infected cells unstimulated or stimulated with 100 ng/mL of TNF-α were used as negative and positive controls, respectively. Cells were lysed with M-PER mammalian extraction reagent (Thermo Scientific) containing protease inhibitors (RPI). One hundred microgram whole cell protein extracts was resolved by SDS-PAGE in 10% acrylamide gels and transferred to nitrocellulose membranes. Blots were incubated with 5% non-fat dry milk in PBS overnight at 4°C and then probed with the antibodies indicated in the Figures, followed by incubation with appropriate Dye-light fluorescent conjugate secondary antibodies. The membranes were scanned with the Odyssey infrared imaging system (Li-Cor). Densitometric analysis was performed using ImageJ® with expression levels of selected target proteins being normalized to those of the housekeeping gene β-actin.

The effect of SVA 3C^pro^ on apoptotic pathways was assessed under transient expression experiments. H1299 cells cultured in 6-wells plates were transfected with 2 μg of pcDNA-Flag-3C and harvested at 18 h post-transfection. Positive control cells were stimulated with TNF-α (100 ng/mL) for 1 h. In the experiments with catalytic dead mutants of SVA 3C^pro^, H1299 cells were transfected with 2 μg of pcDNA-Flag-3C, pcDNA-Flag-3C(H47D), or pcDNA-Flag-3C(C159R) and harvested 18 h post-transfection. Protein extracts and western blots were performed as described above.

To assess the expression of SVA 3C^pro^ during viral replication and its relationship with the cleavage of NF-κB-p65 and apoptosis, STu cells were infected with SVA (MOI = 10) and collected the total protein extract at 2, 4, 5, 6, 7, 8, and 12 h p.i. Western blot was performed as above, and the blots were probed with antibodies indicated in the Figures.

To determine the cleavage site of NF-κB-p65, semi-confluent H1299 cells were cultured in 6-well plates and co-transfected with an empty plasmid (negative control) or pcDNA-Flag-3C (2 μg/well) and either pCMV-HA-NF-κB-p65, pCMV-HA-NF-κB-p65(478R/479D), pCMV-HA-NF-κB-p65(482R/483D), pCMV-HA-NF-κB-p65(478R-483D), pCMV-HA-NF-κB-p65(444L-450R), pCMV-HA-NF-κB-p65(464V-467E), pCMV-HA-NF-κB-p65(444L-450R 478R-483D), or pCMV-HA-NF-κB-p65(464V-467E 478R-483D) (1 μg of each). Cells were harvested at 18 h post-transfection and protein extracts and western blots performed as described above. Antibodies used are indicated in **Figures 8B–D**.

To investigate if caspases or SVA 3C^pro^ were responsible for NF-κB-p65 cleavage, semi-confluent H1299 cells were cultured in 6-well plates and co-transfected with an empty plasmid (negative control) or pcDNA-Flag-3C (1 μg/well) and pCMV-HA-NF-κB-p65 (2 μg/well). At 3 h post-transfection, fresh media was added to negative controls and fresh media containing or not 150 μg/mL of Z-VAD-FMK was added to the cells transfected with pcDNA-Flag-3C and pCMV-HA-NF-κB-p65. Cells were harvested at 18 h post-transfection and protein extracts and western blots performed as described above. Antibodies used are indicated in **Figure 8E**.

### Luciferase Reporter Assays

The effect of SVA on NF-κB-mediated transcription was investigated by luciferase reporter assays. To detect the ability of SVA to inhibit NF-κB activation, semi-confluent H1299 cells cultured in 24-well plates were co-transfected with the NF-κB-luciferase reporter plasmid (315 ng/well) and the control plasmid pRL-TK (35 ng/well) using Lipofectamine 3000 (Life Technologies) and infected with SVA (MOI = 5). SVA-infected cells were stimulated or not with TNF-α (100 ng/mL) at 2, 4, 8, or 12 h p.i. and incubated for 12 h post-TNF-α stimulation. Mock-infected cells were also stimulated with TNF-α for 12 h. The dual-luciferase reporter system (Promega) was used to determine the luciferase activity in each treatment condition following the manufacturer's instructions. Firefly luciferase activity was normalized to renilla luciferase activity and fold-changes calculated based on the levels of luciferase detected in mock-infected/non-stimulated cells.

The effect of SVA 3C^pro^ on NF-κB-mediated transcription was investigated by luciferase assays. H1299 cells were co-transfected with pNF-κB-luciferase reporter plasmid (315 ng/well), pRL-TK (35 ng), and either pcDNA-Flag-3C (550 ng/well), or control plasmids pCMV-Flag-BAP (550 ng/well) or pcDNA-HA-VP1 (550 ng/well). At 14 h post-transfection cells were stimulated with TNF-α, harvested at 12 h post-TNF-α-stimulation and the luciferase activity was determined as described above.

To assess whether the proteolytic activity of 3C^pro^ is required for its effect on NF-κB-mediated transcription, H1299 cells were co-transfected with pNF-κB-luciferase reporter plasmid (315 ng/well), pRL-TK (35 ng), and either pcDNA-Flag-3C (550 ng/well), pcDNA-Flag-3C(H47D) (550 ng/well), or pcDNA-Flag-3C(C159R) (550 ng/well) expression plasmids. At 14 h post-transfection, cells were stimulated with TNF-α, harvested at 12 h post-TNF-α-stimulation and the luciferase activity was determined as described above.

### Site-Direct Mutagenesis

Mutations in the catalytic triad of SVA 3C^pro^ and in the porcine NF-κB-p65 were introduced in the SVA 3C^pro^- and NF-κB-p65-encoding plasmids using the Q5® Site-Directed Mutagenesis Kit (New England BioLabs® Inc.) according to the manufacturer's instructions. Site directed mutagenesis primers containing nucleotide substitutions targeting amino acids H47 and C159 leading to substitutions of H to D, or C to R on SVA 3C^pro^ were designed and used with the site-direct mutagenesis protocol to generate the mutant plasmids pcDNA-Flag-3C(H47D) and pcDNA-Flag-3C(C159R). NF-κB-p65-specific primers containing nucleotide substitutions targeting the putative SVA 3C^pro^ cleavage sites individually (478QL and 482QG) or simultaneously 478QLLNQG were designed and used with the site-direct mutagenesis protocol, resulting in plasmids pCMV-HA-NF-κB-p65(478R/479D), pCMV-HA-NF-κB-p65(482R/483D), and pCMV-HA-NF-κB-p65(478R-483D), respectively. Primers containing nucleotide substitutions targeting putative caspase cleavage sites in NF-κB-p65 coding sequence (444LQFDTDED and 464VFTD) were designed and used with site directed mutagenesis kit, resulting in plasmids pCMV-HA-NF-κB-p65(444L-450R) and pCMV-HA-NF-κB-p65(464V-467E). Double caspase and SVA 3C^pro^ cleavage site-mutants were also generated, resulting in plasmids pCMV-HA-NF-κB-p65(444L-450R 478R-483D) and pCMV-HA-NF-κB-p65(464V-467E 478R-483D). The sequences of each NF-κB-p65 mutant are indicated in **Figure 8E**. Correct nucleotide substitutions were confirmed by DNA sequencing.

### Three-Dimensional Protein Structure Prediction

The SVA 3C^pro^ template-based protein structure modeling was performed using RaptorX web server (74). The best template for SVA 3C^pro^ was the 2wv4A [Foot-and-Mouth disease virus 3C, p-value 1.48e-09 (75)]. SVA 3C^pro^ structure prediction was designed using the PyMOL Molecular Graphics System, version 2.0 Schrödinger, LLC.

### Plaque Assay

Confluent primary STu cells cultured in 6-well plates were infected with serial 10-fold dilutions of SVA (titer 10^7.88^). After 1 h adsorption, the inoculum was removed and fresh 1%-agarose media containing or not 50 μg/mL of Z-VAD-FMK was used to overlay the cells. The agarose overlay was removed and cells were fixed at 24 h p.i. with 3.7% formaldehyde in PBS (pH 7.2) for 20 min. The cells were stained with 1% Crystal Violet solution for 20 min and washed four times with PBS. The area in mm^2^ of each viral plaque was calculated using ImageJ®.

### Statistical Analysis

The average of three independent experiments is presented where appropriate. Error bars represent standard error of the mean (±SEM). Statistical significance of the data was assessed using the *Students' t-*test, Mann-Whitney *U*-test or *Sidak's* multiple comparisons test. The level of statistical significance was defined as *p* < 0.05.

## Results

### SVA Infection Induces Apoptosis *in vitro* and *in vivo*

**Figure 1:**
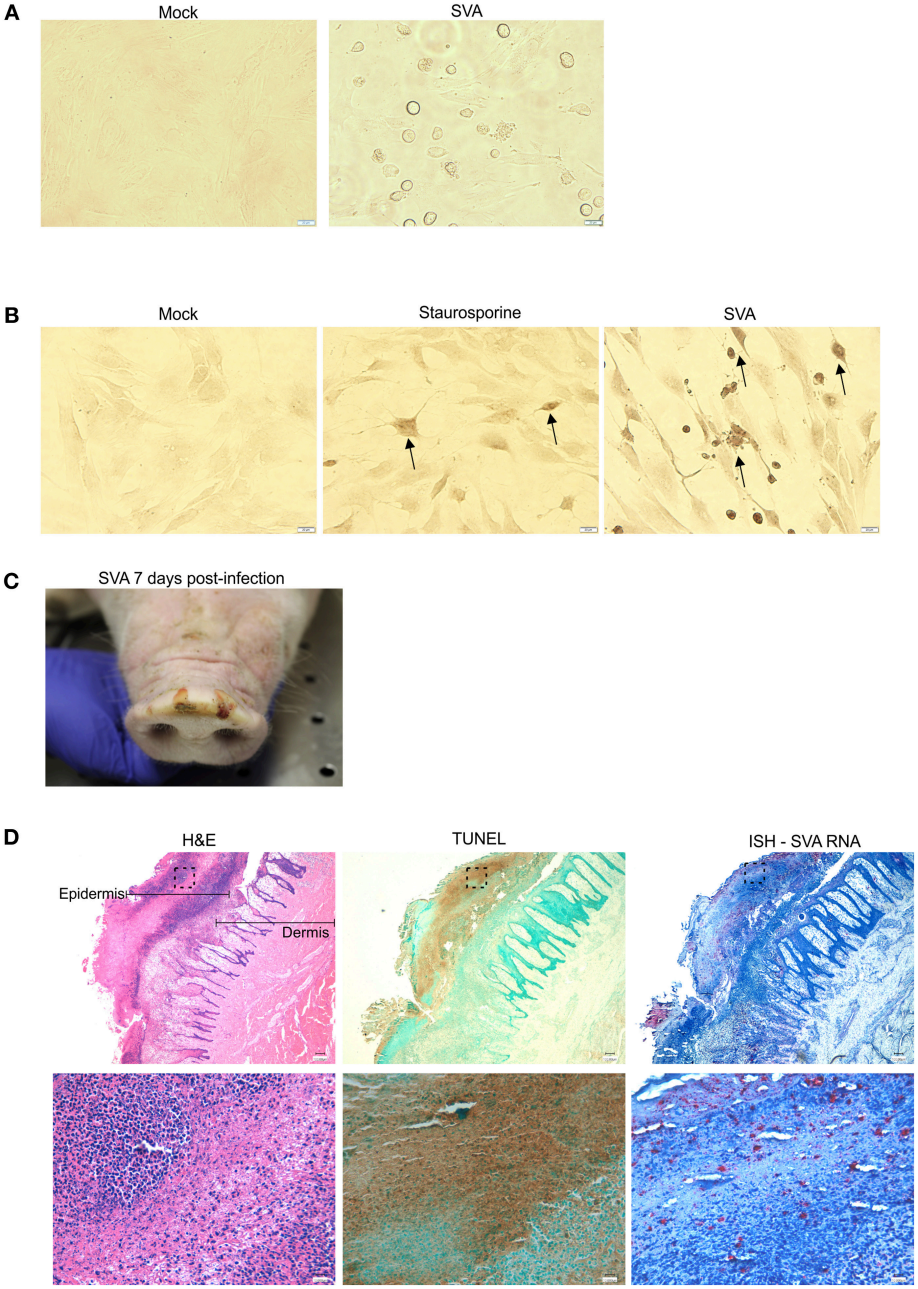
SVA induces apoptosis *in vitro* and *in vivo* **(A)** Cytopathic effect (CPE) caused by SVA in cell culture. Mock-infected (left panel) or SVA-infected (MOI = 5, right panel) primary swine turbinate (STu) cells. 40× magnification. **(B)** TUNEL staining of mock-infected- (left panel), staurosporine-treated- (middle panel) and SVA infected (right panel, MOI = 5) STu cells. 40× magnification. The black arrows indicate apoptotic cells. **(C,D)** Apoptosis occurs during SVA infection in the swine host *in vivo*. Sequential paraffin-embedded skin lesions collected from SVA-infected pigs on day 7 post-infection **(C)** were subjected to hematoxylin and eosin staining (**D**, left panels), TUNEL (**D**, middle panels), and in situ hybridization for detection of SVA RNA (RNAScope®) (**D**, right panels). 4× (top panels) and 40× (bottom panels) magnifications. Areas highlighted in the top panels are represented in the bottom higher magnification images.

Replication of SVA in primary STu cells induces cytopathic effect (CPE) characterized by cell rounding and plasma membrane blebbing (Figure 1A). Therefore, induction of apoptosis was investigated during SVA infection. Initially, TUNEL was performed in SVA-infected cells to detect exposed 3′-OH ends of DNA fragments, which are generated in response to apoptotic signals. As shown in Figure 1B, apoptotic nuclei were detected in SVA-infected cells and cells treated with staurosporine (a potent inducer of apoptosis) (arrows), suggesting induction of apoptosis during SVA infection.

To assess whether apoptosis occurs during viral infection in the swine host *in vivo*, paraffin-embedded skin sections collected from SVA-infected pigs on day 7 post-infection (p.i.), were subjected to TUNEL. Skin sections were obtained from characteristic SVA vesicular lesions (Figure 1C). Sequential skin sections were subjected to H&E staining, ISH to detect SVA RNA and TUNEL to detected apoptosis. As shown in Figure 1D, strong brown staining indicating the presence of apoptotic cells was detected by TUNEL (Figure 1D, middle panels), in an area that presented histological changes caused by SVA replication in the dermis and epidermis (Figure 1D, left panels), and that coincided with the ISH staining for SVA RNA (Figure 1D, right panels).

**Figure 2:**
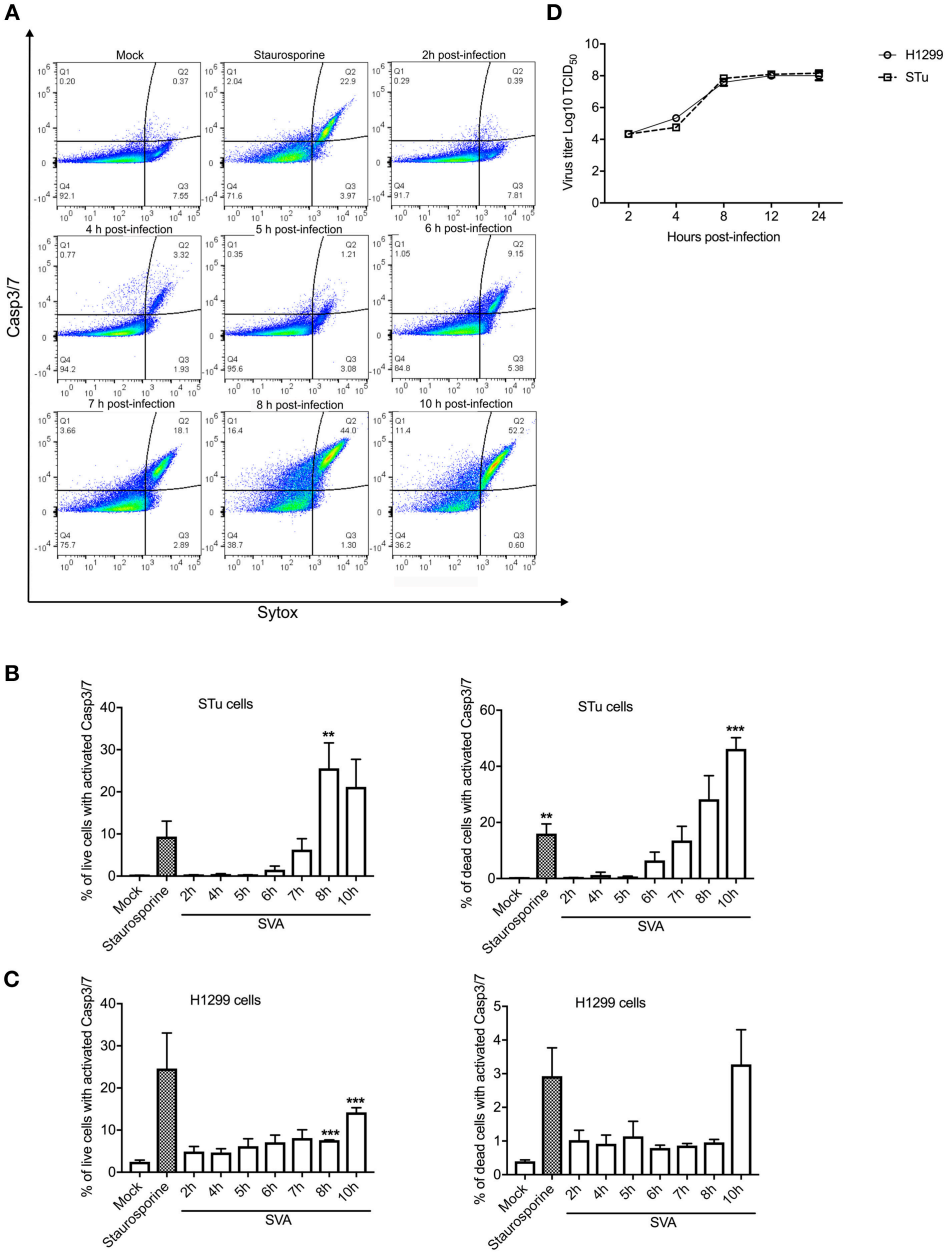
Activation of caspases 3/7 (Casp-3/-7) during SVA infection. **(A)** Gating strategy used to determine percent of cells with active Caspase-3/7 and stained with Sytox-green during SVA infection. Caspase activation was determined using the CellEventTM Caspase-3/7 Green Flow Cytometry Assay kit. **(B**,**C)** Caspase-3/7 activation in STu or H1299-infected cells (MOI = 5) negative (left panel) or positively (right panel) stained with Sytox-green, representing live cells (early apoptotic cells), or dead cells (late apoptotic cells). The flow cytometry data were acquired with an Attune NxT flow cytometer and analyzed using FlowJo software. The results represent the average of three independent experiments in STu **(B)** or H1299 **(C)** cells. Error bars represent SEM calculated based on the results of the independent experiments; **p < 0.05 and ***p < 0.01, compared to mock-infected cells. **(D)** Single-step growth curves of SVA (MOI = 5) in STu or H1299 cells. Cells were collected at indicated time points and virus titers expressed as log_10_ tissue culture infections dose 50 (TCID_50_) per milliliter. The results represent the average of three independent experiments. Error bars represent SEM.

Since Casp-3/-7 are the main executioners of apoptosis, and are responsible for the proteolytic cleavage of many cellular proteins during cell death (57), we assessed their activation during SVA infection by flow cytometry. SVA induced activation of Casp-3/-7 from 7 h p.i. onward in STu cells, with an increasing number of cells containing active caspases detected until 10 h p.i. (Figures 2A,B). Notably, the frequency of live cells with active Casp-3/-7 representing cells in early stages of apoptosis were ~4-fold higher at 8 h p.i. (25.56%; *p* < 0.05) than those detected at 7 h p.i. (6.29%, when compared to mock-infected cells) (Figure 2B). Interestingly, at 10 h p.i., the percentage of active Casp-3/-7 detected in Sytox-green positive cells, indicating late stages of apoptosis, reached 46.23% (*p* < 0.01 when compared to mock-infected cells). A significant increase in the frequency of cells presenting active Casp-3/-7 was also detected at 8–10 h p.i. (7.6–14.2%, *p* < 0.01 when compared to mock-infected cells) in SVA-infected H1299 cells (Figure 2C). Similar to the results in STu cells, the frequency of late-stage apoptotic cells (Casp-3/7 and Sytox positive cells) was markedly increased at 10 h p.i. in H1299 cells. These results demonstrate that Casp-3/-7 are activated between 7 and 10 h post-SVA infection, which corresponds to the time in which the virus completes one round/cycle of replication (Figure 2D). Together these results demonstrate that SVA infection induces apoptosis late during infection *in vitro* and in skin lesions in the natural swine host *in vivo*.

### SVA Infection Modulates Expression of Pro-Inflammatory and Apoptosis Related Genes

**Figure 3:**
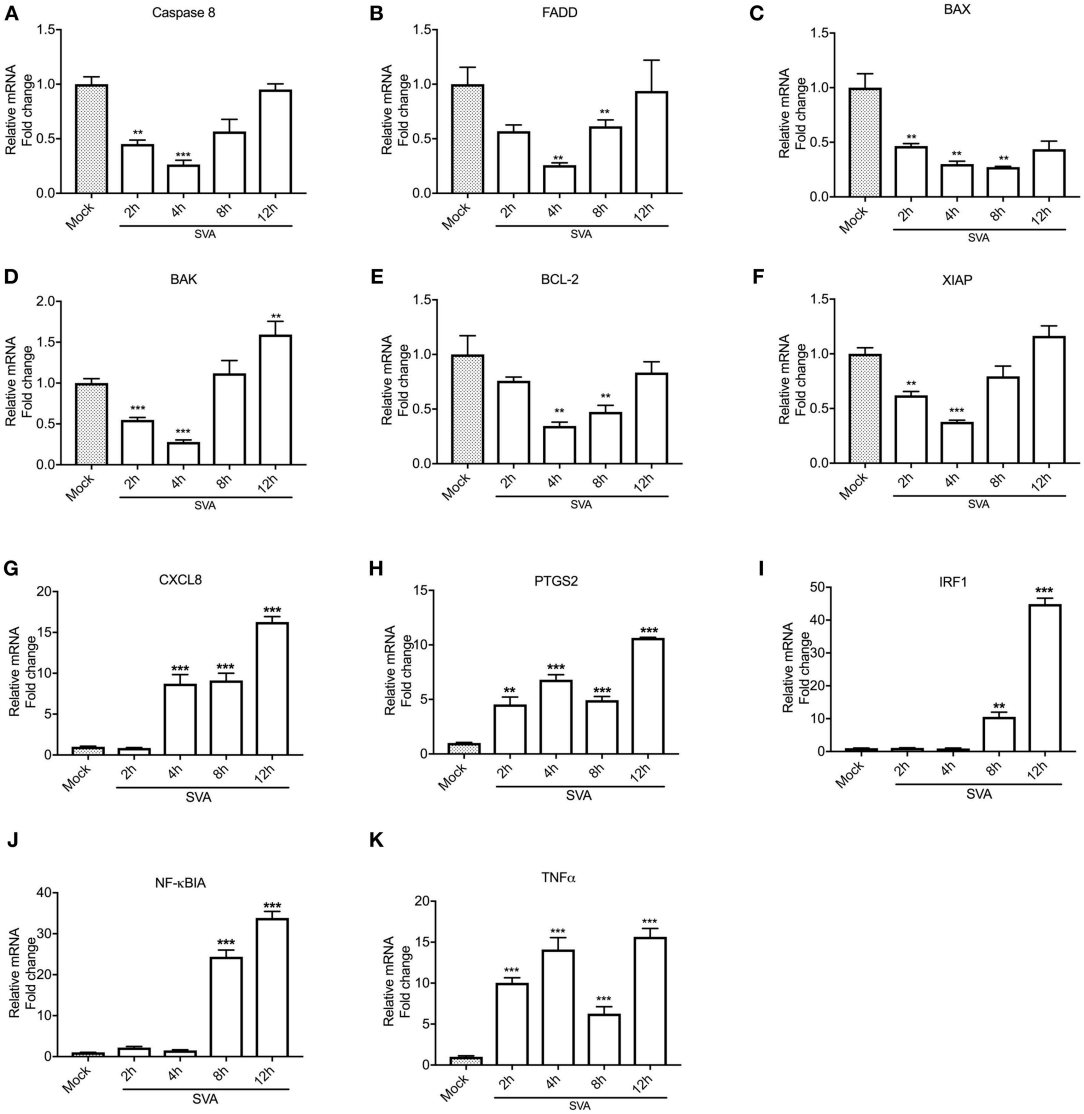
Expression levels of select NF-κB target genes during SVA infection. Expression of pro-apoptotic (Caspase 8, FADD, BAX, and BAK), anti-apoptotic (BCL-2 and XIAP) and pro-inflammatory (CXCL8, PTGS2, IRF1, NFκBIA, and TNF-α) genes were assessed by RT-qPCR at different time points post-SVA infection in primary swine turbinate (STu) cells (2, 4, 8, and 12 h; MOI = 5). Expression levels of Caspase 8 **(A)**, FADD **(B)**, BAX **(C)**, BAK**(D)**, BCL-2 **(E)**, XIAP **(F)**, CXCL8 **(G)**, PTGS2 **(H)**, IRF1 **(I)**, NFκBIA **(J)**, and TNF-α **(K)** were normalized to that of Glyceraldehyde 3-phosphate dehydrogenase (GAPDH). Mock-infected cells were used as negative control. The results were expressed as relative fold changes in mRNA levels compared to mock-infected cells. The results represent the average of three biological replicates. Error bars represent SEM, **p < 0.05 and ***p < 0.01, compared to mock-infected cells.

NF-κB is one of the major players modulating host cell apoptosis and pro-inflammatory responses (76, 77). Thus, we assessed transcription of NF-κB-regulated genes during SVA infection. For this, STu cells were mock-infected or infected with SVA (MOI = 5) and total RNA was extracted during infection (2, 4, 8, and 12 h). As shown in Figures 3A–F, all pro- (Casp-8, FADD, BAX, and BAK) or anti-apoptotic (BCL-2 and XIAP) genes tested were down regulated at early time points post-SVA infection (2–4 h p.i.) (*p* < 0.05 when compared to mock-infected cells). Additionally, the pro-inflammatory NF-κB-regulated genes, including CXCL8, PTGS2, IRF1, NF-κBIA, and TNF-α were up-regulated during SVA infection (4–12 or 8–12 h p.i.) (Figures 3G–**K**). These results suggest modulation of NF-κB-regulated gene expression during SVA infection in primary STu cells.

### Effect of SVA Infection on the NF-κB Signaling Pathway

**Figure 4:**
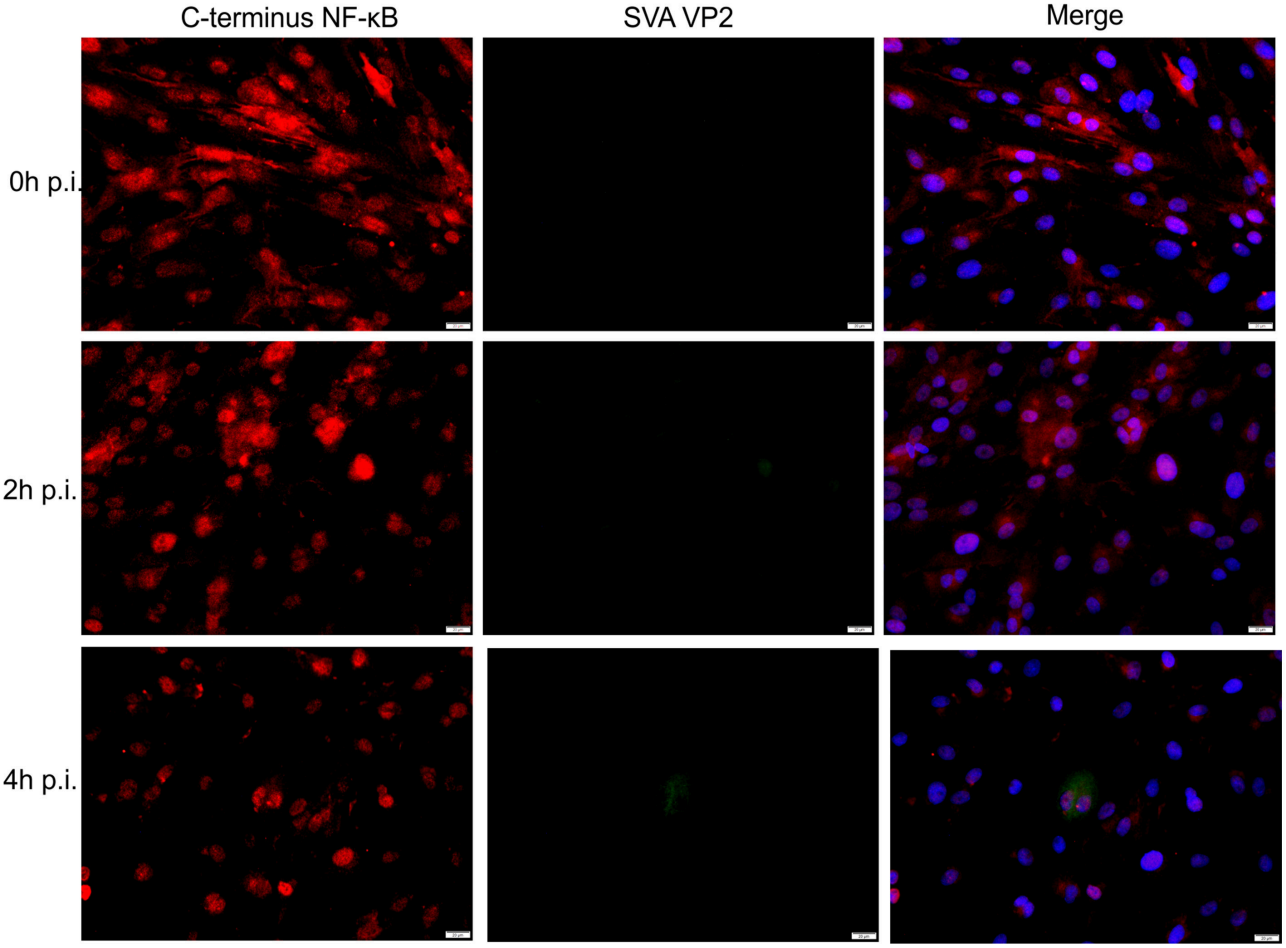
Effect of SVA on expression of NF-κB-p65 in STu cells in early stages of infection. Primary swine turbinate (STu) cells were infected with SVA (MOI = 5) and stained with a SVA VP2 monoclonal antibody (middle panel; green—Alexa fluor 488) and a rabbit polyclonal antibody specific for C-terminus of NF-κB-p65 (left panel; red—Alexa fluor 594). The cell nuclei were stained with DAPI (right panel; blue). 40× magnification.

Given the transcriptional changes and modulation of apoptotic pathway observed in SVA infected cells (Figures 2 and 3), we investigated activation of NF-κB signaling during SVA infection. Since NF-κB-p65 is one of the key NF-κB subunits, that is directly responsible for the transactivation of NF-κB target genes (78), expression and activation of NF-κB-p65 were assessed in SVA infected cells using IFA. As shown in Figure 4, at ~2 h p.i. NF-κB-p65 translocated to the nucleus of cells, indicating activation of the NF-κB pathway following SVA infection.

**Figure 5:**
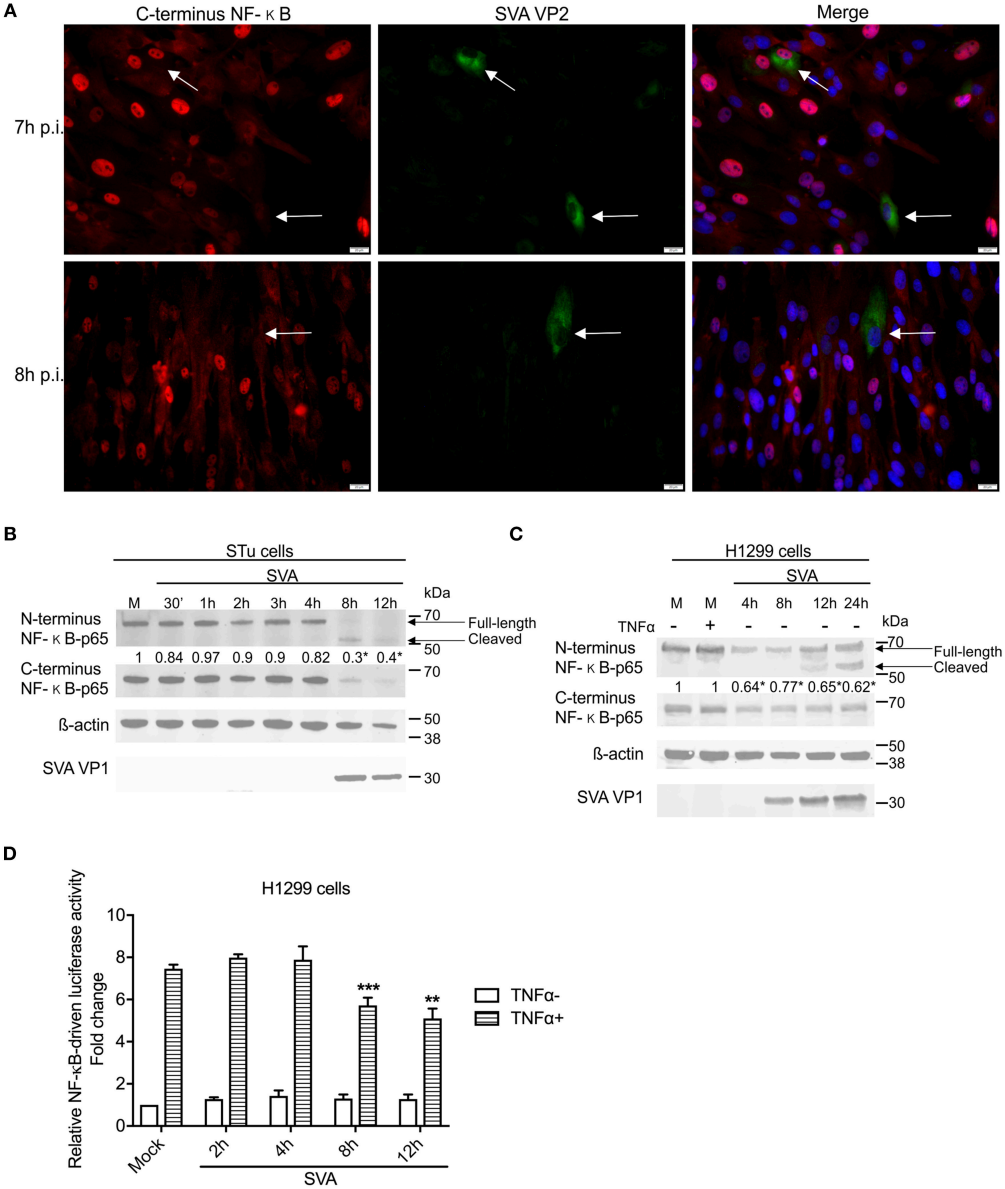
NF-κB-p65 is cleaved during SVA infection. **(A)** Immunofluorescence of primary swine turbinate (STu) cells infected with SVA (MOI = 1) and stained with a monoclonal antibody specific for SVA VP2 (middle panel; green—Alexa fluor 488) and a rabbit polyclonal antibody specific for C-terminus of NF-κB-p65 (left panel; red—Alexa fluor 594). The cell nuclei were stained with DAPI (right panel; blue). 40× magnification. The white arrows indicate the cells infected with SVA. **(B,C)** Western blot analysis of primary STu or H1299 showing cleavage of NF-κB-p65 during SVA infection. Total protein extracts were analyzed by western blot using antibodies specific to N-terminus NF-κB-p65, C-terminus NF-κB-p65, SVA VP1, or β-actin. Densitometric analysis was performed to quantify the levels of C-terminus NF-κB-p65. The densitometry of C-terminus NF-κB-p65 bands were normalized to the β-actin control and expressed as relative densitometry to mock infected control cell (numbers on top of NF-κB-p65 blot; *p < 0.05; compared to mock-infected cells). The results are representative of four independent experiments. **(D)** Luciferase reporter assays in H1299 cells during SVA infection. Cells were infected and stimulated with TNF-α at the indicated time points. Firefly luciferase activity was determined at 12 h post-TNF-α stimulation and normalized to the renilla luciferase activities. The results were expressed as relative fold changes in luciferase activity (**p < 0.05 and ***p < 0.01, compared to mock-infected TNF-α-stimulated cells). Error bars represent SEM calculated based on the results of the three independent experiments.

Interestingly, at later times p.i. (7–8 h p.i.) on a low MOI experiment, many of the SVA-infected cells presented a marked decrease in expression of NF-κB-p65 (Figure 5A), suggesting potential degradation or cleavage of the C-terminus region of this transcription factor. To assess whether the decreased levels of NF-κB-p65 observed in SVA-infected cells at late times p.i. occurred due to degradation or cleavage of the C-terminus of the molecule, expression of NF-κB-p65 was evaluated throughout the virus infection cycle using western blots. As shown in Figure 5B, similar levels of N- and C-terminus NF-κB-p65 were detected in SVA infected cells up to 4 h p.i., when compared to control mock-infected cells. At 8 h p.i., a marked decrease in the levels of NF-κB-p65 (~70% compared to the average of mock cells) was observed in SVA-infected cells (*p* < 0.05; Figure 5B). Notably, accumulation of a cleaved product of ~50 kDa, corresponding to N-terminus of NF-κB-p65 was detected at 8–12 h p.i. (Figure 5B). Similar results were observed in the human carcinoma cell line H1299 (Figure 5C).

NF-κB transcriptional activity was assessed during SVA infection using an NF-κB-luciferase reporter assay. A significant decrease in TNF-α-induced NF-κB-mediated luciferase activity was observed between 8 and 12 h post-SVA infection (Figure 5D). Collectively, these results demonstrate a fine modulation of NF-κB pathway during SVA infection. While early during infection the NF-κB pathway is activated, at later times p.i., SVA infection induces cleavage of NF-κB-p65, which results, in lower transcriptional activity of this important modulator of host cell inflammation and survival.

### Expression of SVA 3C^pro^ Is Associated With Apoptosis and Cleavage of NF-κB-p65

**Figure 6:**
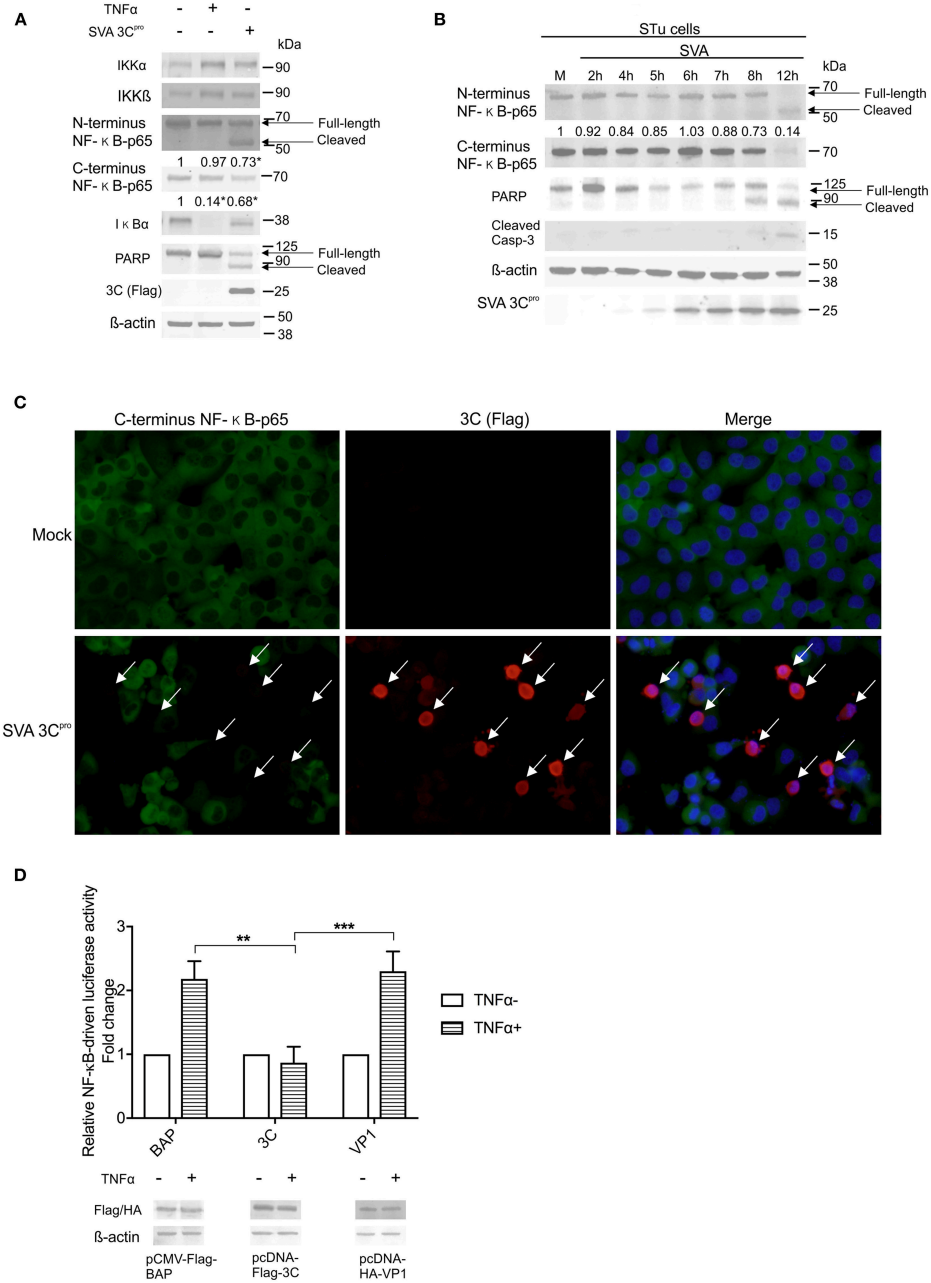
Expression of SVA 3Cpro is associated with apoptosis and cleavage of NF-κB-p65. **(A)** Western blot analysis of H1299 cells transiently expressing SVA 3Cpro demonstrating cleavage of NF-κB-p65 and PARP. Non-transfected cells were stimulated or not with TNF-α for 1 h, and used as negative and positive controls, respectively. Total protein extracts were analyzed by western blots using antibodies indicated on the left. The results are representative of three independent experiments. **(B)** Western blot analysis of STu cells demonstrating cleavage of NF-κB-p65 and PARP during SVA infection. Total protein extracts were analyzed using antibodies indicated on the left. Densitometric analysis was performed to quantify the levels of C-terminus NF-κB-p65. The densitometry of C-terminus NF-κB-p65 bands were normalized to the β-actin control and expressed as relative densitometry to mock infected control cells. **(C)** Immunofluorescence staining showing decreased levels of NF-κB-p65 in in SVA 3Cpro expressing H1299 cells. Flag-tag antibody (red—Alexa fluor 594) and rabbit polyclonal antibodies specific for C-terminus NF-κB-p65 (green—Alexa fluor 488). The cell nuclei were stained with DAPI (blue). 40× magnification. The white arrows indicate the cells expressing SVA 3Cpro (red). **(D)** Luciferase reporter assays in H1299 cells expressing SVA 3Cpro. At 14 h post-transfection of luciferase and 3Cpro expressing plasmids, cells were stimulated with TNF-α for 12 h (TNFα+) or left unstimulated (TNF α-). Luciferase activity was determined, and firefly luciferase activity normalized to renilla luciferase activity. The results were expressed as relative fold changes in luciferase activity (**p < 0.05; ***p < 0.01; compared Flag-BAP and HA-VP1 expressing cells). Data shown represent the average of five independent experiments. Error bars represent SEM calculated based on the results of the independent experiments. Western blot showing expression of BAP, SVA 3Cpro, and VP1 in samples examined by luciferase assays (bottom panel).

Next we sought to dissect the mechanisms underlying SVA-induced host-cell apoptosis and its link with cleavage of NF-κB-p65. First, we assessed the potential role of SVA non-structural protein 3C^pro^, the main protease encoded by the virus (1), on apoptosis and cleavage of NF-κB-p65 using western blots. Expression of IKKα, IKKβ, IκBα, and NF-κB-p65 were investigated. Additionally, cleavage of PARP, a hallmark of host cell apoptosis (79), was also assessed in 3C^pro^ expressing cells. While expression of 3C^pro^ did not affect expression levels of the upstream NF-κB kinases (IKKα, IKKβ) (Figure 6A), a marked decrease in the levels of IκB-α and NF-κB-p65 were observed in 3C^pro^ expressing cells (Figure 6A). Accumulation of the cleaved 50 kDa-NF-κB product, observed in SVA-infected cells (Figures 5B,C), was also detected in 3C^pro^ expressing cells, when protein extracts were probed with the antibody against N-terminus NF-κB-p65 (Figure 6A). IFA experiments using an antibody specific for the C-terminus region of NF-κB-p65 confirmed decreased levels of C-terminus NF-κB-p65 in 3C^pro^ expressing cells (Figure 6C). Similar results were observed in primary STu cells (data not shown). Importantly, expression of SVA 3C^pro^ also resulted in cleavage of PARP (Figure 6A), suggesting that the viral protease induces apoptosis when expressed outside the context of SVA infection.

The effect of SVA 3C^pro^ expression on cleavage of NF-κB-p65 and activation of apoptotic pathways in the context of SVA infection were investigated by western blots. Expression of SVA 3C^pro^ was first detected using a SVA 3C^pro^-specific rabbit polyclonal antibody at 5 h p.i., with increasing levels of the protein being detected up to 12 h p.i. (Figure 6B). Decreased levels of C-terminus NF-κB-p65 were observed after 7 h post-SVA infection (0.88) (Figure 6B). In addition, accumulation of the cleaved 50 kDa-NF-κB product was observed at 8 h p.i. (Figure 6B). Notably, a decrease in the levels of the full-length PARP was evident after 5 h p.i., with a cleaved product of the protein accumulating in infected cells after 8 h p.i. (Figure 6B). Detection of cleaved Casp-3 at 8–12 h p.i. confirmed induction of apoptosis late during SVA infection (Figure 6B).

The effect of 3C^pro^ expression on the transcriptional activity of NF-κB was investigated using a luciferase reporter assay. Expression of SVA 3C^pro^ in H1299 cells completely suppressed TNF-α-induced NF-κB-luciferase activity (Figure 6D) as evidenced by significantly decreased luciferase activity in SVA 3C^pro^-expressing cells (~2.2-fold, *p* < 0.05) when compared to cells expressing either VP1- or BAP-control proteins (Figure 6D). These results indicate that expression of SVA 3C^pro^ results in reduced NF-κB-mediated transcription following stimulation of cells with TNF-α. Collectively, these findings suggest that expression of SVA 3C^pro^ could be associated with cleavage of NF-κB-p65 and induction of apoptosis in SVA infected cells.

### The Protease Activity of 3C^pro^ Is Required for Induction of Apoptosis and Cleavage of NF-κB-p65

**Figure 7:**
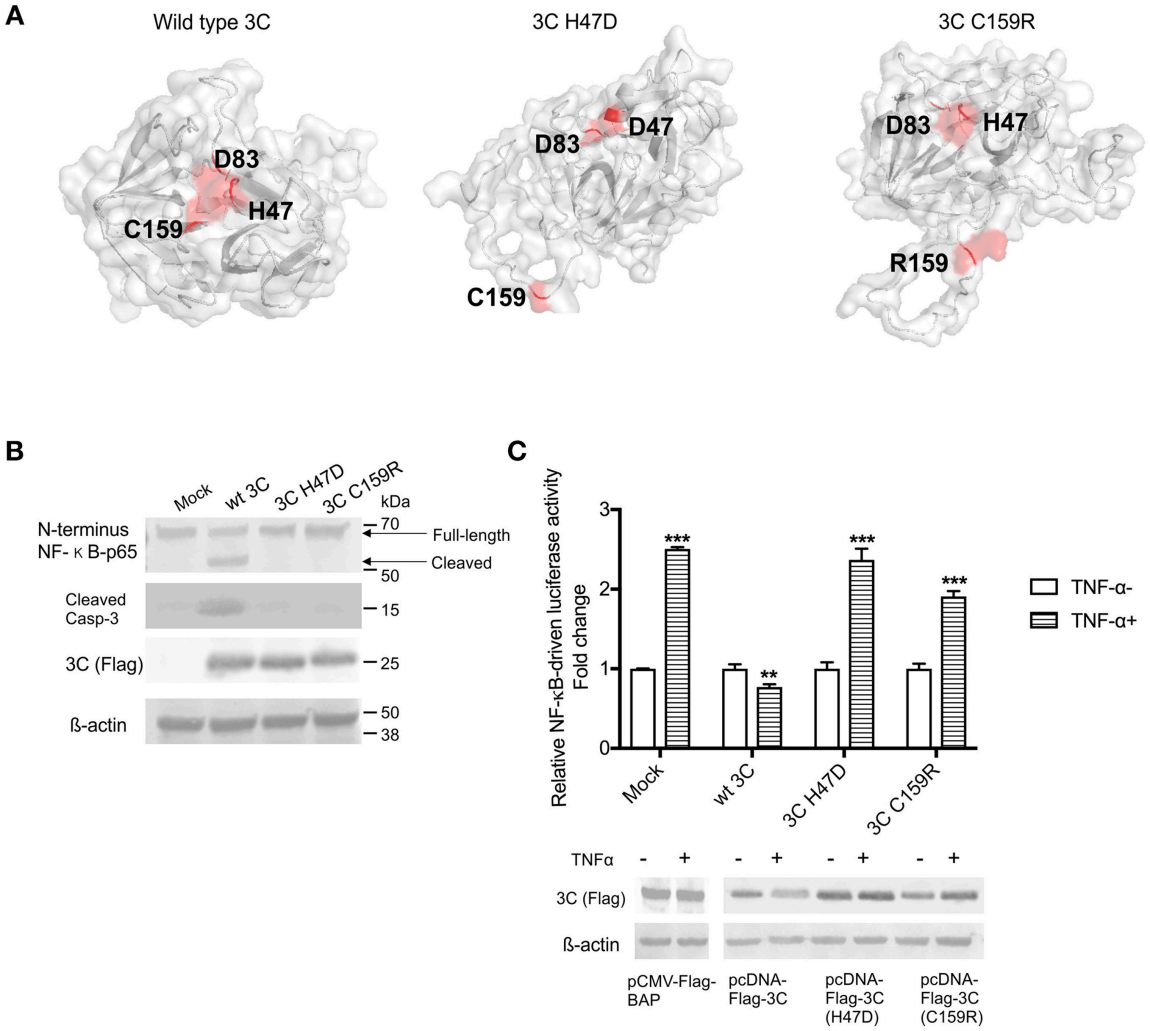
The protease activity of 3C^pro^ is required for induction of apoptosis. **(A)** Three-dimensional protein structure prediction. The wild type SVA 3C^*pro*^ and the 3C^pro^ mutants (H47D and C159R) template-based protein structure models were determined using RaptorX web server (63). Red dots represent the aa present in the catalytic triad (H47, D83, and C159), or the point mutations H47D or C159R introduced in SVA 3C^pro^. **(B)** Western blot analysis of H1299- cells expressing SVA 3C^*pro*^ showing that mutations in the catalytic triad of the protease impairs its pro-apoptotic function. Protein extracts were analyzed by western blot using antibodies specific for N-terminus NF-κB-p65, cleaved Caspase 3, Flag-tag, and β-actin as loading protein control. The results are representative of three independent experiments. **(C)** Luciferase assay showing that SVA 3C^pro^ catalytic triad is important for its inhibitory effect on NF-κB-p65 transcriptional activity. H1299 cells were transfected with NF-κB-luciferase reporter plasmids and either wild type or mutant 3C-expressing plasmids. At 12 h post-transfection cells were stimulated with TNF-α for 12 h, and luciferase activity determined. Firefly luciferase activity was normalized to the renilla luciferase activity. The results were expressed as relative fold changes in luciferase activity (**p < 0.05 and ***p < 0.01, compared to BAP-expressing cells treated with TNF-α). Results shown represent the average of five independent experiments. Error bars represent SEM. Western blots showing expression of BAP, wild type 3C^pro^, 3C H47D, and 3C C159R in samples examined by luciferase assays (bottom panel).

To assess whether the protease activity of SVA 3C^pro^ contributes to induction of apoptosis and/or cleavage of NF-κB-p65, we constructed two plasmids encoding for mutants of SVA 3C^pro^. The picornavirus 3C^pro^ contains a conserved catalytic triad (His, Asp, Cys) that is required for its protease activity (4, 80). To disrupt the catalytic triad and, consequently, the proteolytic activity of SVA 3C^pro^ we introduced a mutation at His 47 (H47D) or Cys 159 (C159R) using site-directed mutagenesis (Figure 7A). The effect of these mutations on apoptosis and cleavage of NF-κB-p65 were investigated in 3C^pro^-expressing cells. H1299 cells were transfected with plasmids encoding the wild type or each of the 3C^pro^ mutants (H47D or C159R) and total protein extracts were subjected to western blot analysis. Notably, while expression of the wild type SVA 3C^pro^ resulted in activation of Casp-3 and cleavage of NF-κB-p65, expression of the 3C^pro^ catalytic dead mutants H47D or C159R did not lead to Casp-3 activation nor NF-κB-p65 cleavage (Figure 7B).

The effect of SVA 3C^pro^ protease activity on NF-κB-mediated gene transcription was investigated using luciferase reporter assays. Notably, while expression of the wild type SVA 3C^pro^ markedly inhibited TNF-α-induced NF-κB-mediated luciferase activity (*p* > 0.05), expression of the 3C^pro^ mutants (H47D and C159R) did not inhibit TNF-α-induced NF-κB-mediated luciferase activity (Figure 7C). These results suggest that the protease activity of SVA 3C^pro^ may be the trigger that leads to host cell apoptosis and potentially to cleavage of NF-κB-p65 in host cells.

### Assessing the Mechanism of NF-κB-p65 Cleavage

**Figure 8:**
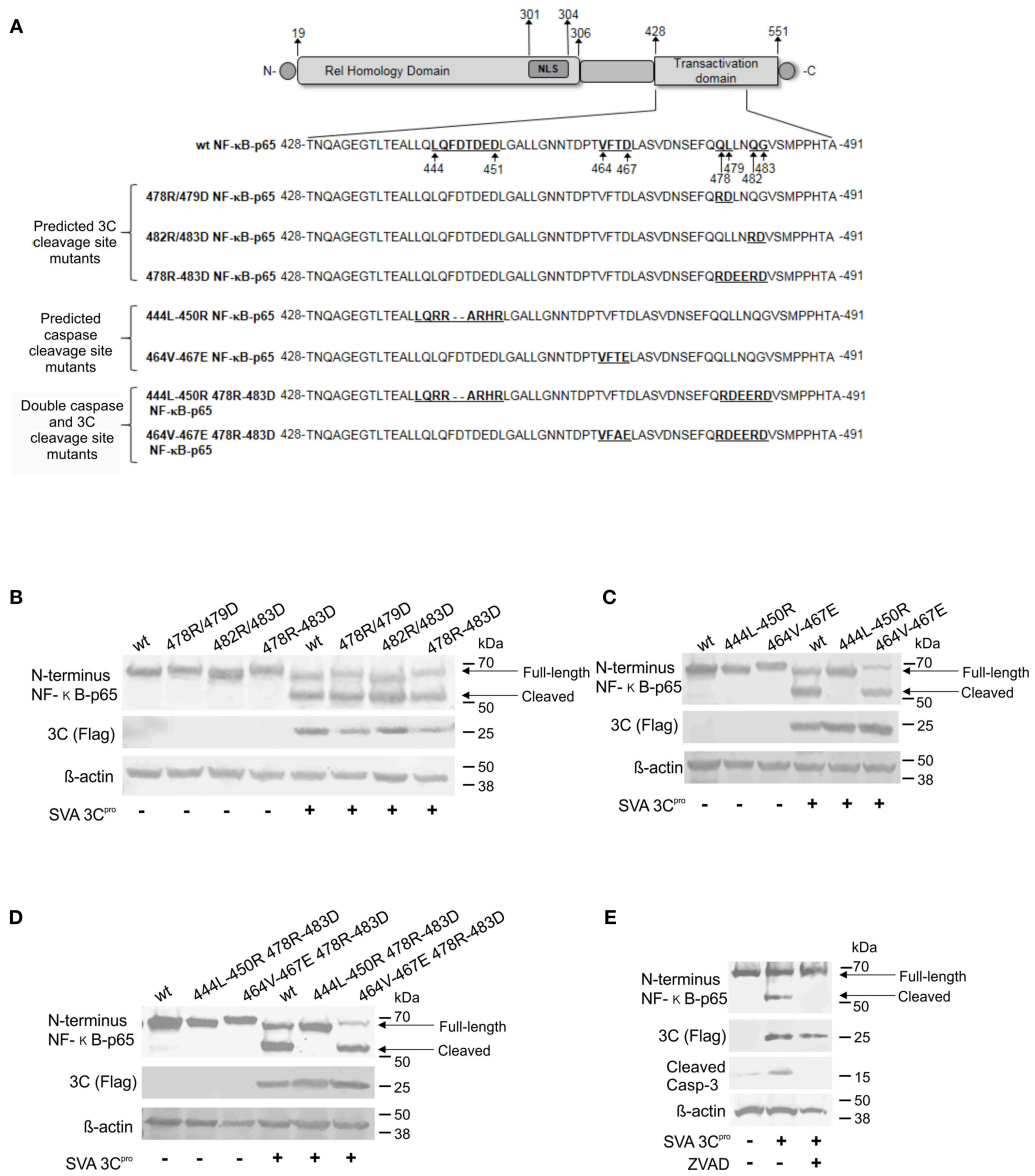
Cleavage of NF-κB-p65 is mediated by caspases and not by the direct action of SVA 3C^pro^. **(A)** Schematic representation of NF-κB-p65 protein depicting predicted cleavage sites for Casp-3/-6 (81) and/or SVA 3C^pro^ in the TAD. Western blot analysis of H1299 cells expressing wild type or mutant NF-κB-p65 proteins containing aa substitutions on SVA 3C predicted cleavage sites **(B)**, caspase predicted cleavage sites **(C)**, or double SVA 3C^pro^ and caspase predicted cleavage sites **(D)**. **(E)** Western blot to assess cleavage of NF-κB-p65 in SVA 3C^pro^-expressing cells in the presence or absence of caspase inhibitor Z-VAD-FMK. Blots in **(B–E)** were probed with antibodies indicated on the left.

To dissect whether cleavage of NF-κB-p65 is directly mediated by SVA 3C^pro^ or whether it is secondary to caspase activation following 3C^pro^ expression, we generated a panel of plasmids encoding NF-κB-p65 proteins containing mutations on predicted cleavage sites for Casp-3/-6 (81) and/or for the SVA 3C^pro^ (Figure 8A). Two cleavage sites for 3C^pro^ (Q/L or Q/G, 478QL, and 482QG) (Figure 8A) and two cleavage sites for caspases (444LQFDTDED and 464VFTD) were identified in the transactivation domain (TAD) of NF-κB-p65 (Figure 8A). These predicted cleavage sites were mutated using site directed mutagenesis and resultant plasmids used in transient expression experiments. (Figure 8A). Cleavage of NF-κB-p65 was evaluated after transient co-expression of wild type- or mutant NF-κB-p65 proteins and the SVA 3C^pro^ by western blots. As shown in Figure 8B, all NF-κB-p65 constructs with mutations on predicted cleavage sites for SVA 3C^pro^ were cleaved when co-expressed with the viral protease. Notably, when the NF-κB-p65 containing mutations in the predicted cleavage sites for cellular caspases (444L-450R and 464V-467E) were co-expressed with 3C^pro^, NF-κB-p65 464-467E mutant was cleaved, while no cleavage of NF-κB-p65 444L-450R mutant was observed (Figure 8C). This phenotype was confirmed in double NF-κB-p65 mutants, containing 3C^pro^- and Casp-cleavage site mutations. As shown in Figure 8D, when the mutation 444L-450R in the caspase cleavage site was introduced in NF-κB-p65 containing a mutation on the 3C^pro^ cleavage site (478R-483D), NF-κB-p65 was not cleaved (Figure 8D). Additionally, inhibition of caspases by incubation of cells with Z-VAD-FMK (a pan caspase inhibitor) prevented cleavage of NF-κB-p65 in cells expressing 3C^pro^ (Figure 8E). Together, these results show that NF-κB-p65 cleavage occurs at the caspase cleavage site (444LQFDTDED), suggesting that cleavage of NF-κB-p65 is mediated by caspases and not by the direct action of SVA 3C^pro^.

### Link Between NF-κB-p65 and Apoptosis During SVA Infection

**Figure 9:**
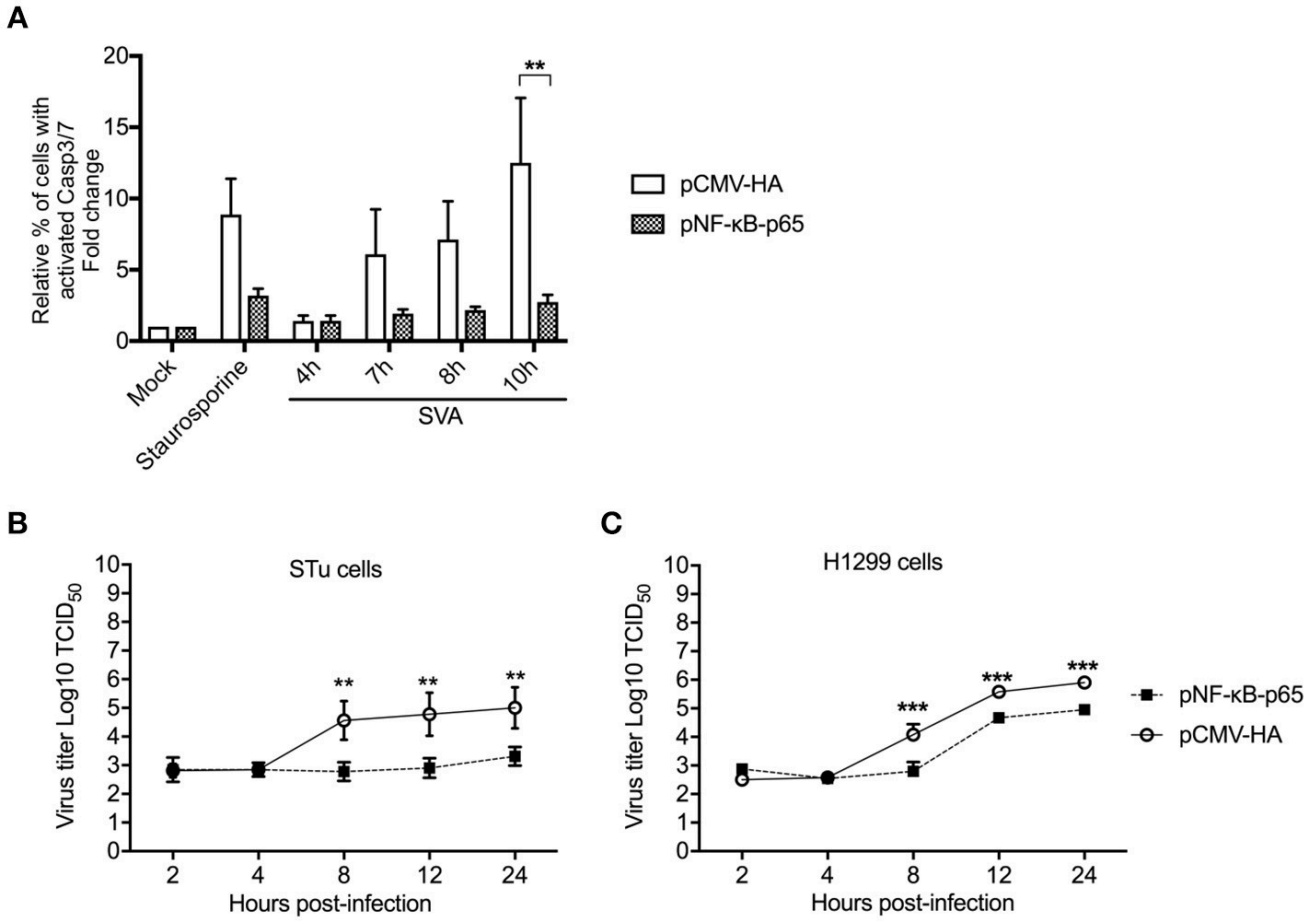
Overexpression of NF-κB-p65 suppresses SVA-induced apoptosis and decreases SVA yields. **(A)** Flow cytometry analysis of STu cells showing that transient expression of NF-κB-p65 decreases apoptosis induced by SVA infection. Cells were stained with CellEvent™ Caspase-3/7 Green Flow Cytometry Assay kit. The flow cytometry data were acquired with an Attune NxT flow cytometer and analyzed using FlowJo software. The results were expressed as percent of cells with active Casp-3/-7. Error bars represent SEM; **p < 0.05 compared to mock-infected cells; Sidak's multiple comparison test. **(B)**, **(C)** Replication kinetics of SVA in STu **(B)** or H1299 cells **(C)** transiently expressing NF-κB-p65. Cells were infected and harvested at indicated time points and virus titers determined by the Spearman and Karber's method and expressed as log_10_ tissue culture infections dose 50 (TCID_50_) per milliliter. The results represent the average of three independent experiments. Error bars represent SEM (**p < 0.05, ***p < 0.01; comparisons between each time point, Students' *t*-test).

Given our results demonstrating the association between cleavage of NF-κB-p65, and host cell apoptosis late in SVA infection, we investigated the importance of this process on virus replication. First, we assessed the effect of NF-κB-p65 on SVA-induced apoptosis using a Casp-3/-7 flow cytometry-based assay in STu cells transiently expressing NF-κB-p65. Notably, expression of NF-κB-p65 resulted in markedly low activation of Casp-3/-7 following SVA infection when compared to cells transfected with an empty control plasmid (Figure 9A). Indeed, when NF-κB-p65 was expressed, caspase activation levels in SVA-infected- or staurosporine-treated cells were similar to those observed in mock-infected cells (Figure 9A). These results indicate that overexpression of NF-κB-p65 prevents SVA-induced apoptosis in STu cells.

Since expression of NF-κB-p65 led to reduced levels of apoptosis in SVA infected cells, we next assessed its effect on SVA replication. Expression of NF-κB-p65 in both STu or H1299 cells resulted in reduced viral yields as evidenced by significantly lower viral titers in cells expressing NF-κB-p65, when compared to cells transfected with the empty control plasmid (*p* < 0.05, STu; *p* < 0.01, H1299) (Figures 9B,C). Together these results suggest that Casp-mediated cleavage of NF-κB-p65 (Figure 8) and induction of host cell apoptosis late during SVA infection (Figure 2) might be critical for virus replication and/or release from infected cells.

### Late Induction of Apoptosis Seems Essential for SVA Infection Cycle

**Figure 10:**
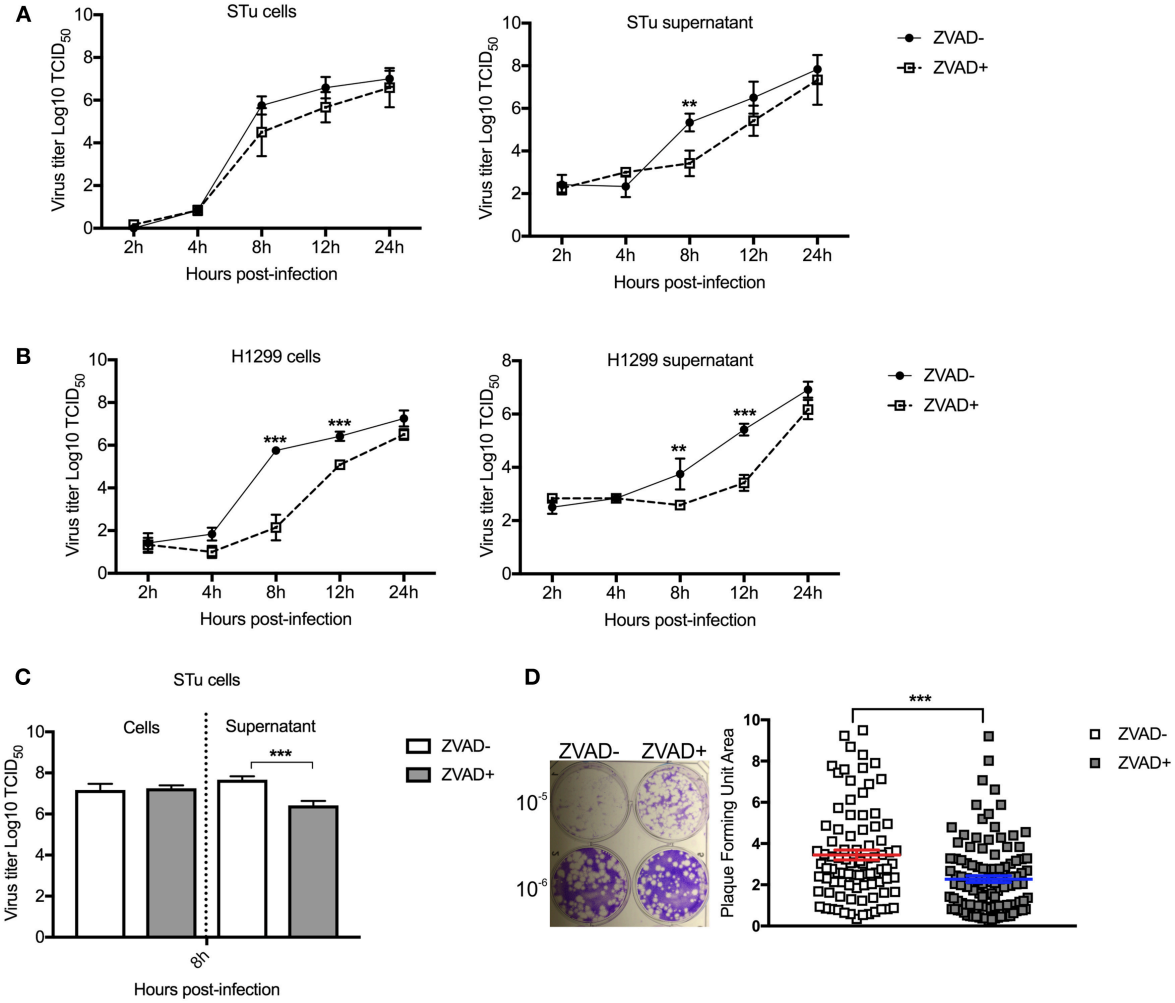
Induction of apoptosis late in SVA infection is important for virus release and/or spread from infected cells. Multiple-step growth curves in STu **(A)** or H1299 **(B)** cells (MOI = 0.1), and single step growth curve STu **(C)** cells (MOI = 5) treated or not with Z-VAD-FMK, a pan-caspase inhibitor. Cells and supernatants were harvested separately at the indicated time points and subjected to virus titration. The virus titers were determined by the Spearman and Karber's method and expressed as log_10_ tissue culture infections dose 50 (TCID_50_) per milliliter. Results represent the average of three independent experiments. Error bars represent SEM **p < 0.05, ***p < 0.01; comparisons between each time point in the multiple-step growth curve; and ***p < 0.01; comparisons between the time point in the single-step growth curve. **(D)** Plaque assay in STu cells treated or not with Z-VAD-FMK showing reduced viral spread in cells in which apoptosis is inhibited. In the right panel, the graphic represents SVA plaque forming unit area of cells treated or not with Z-VAD-FMK (10^−6^ dilution). Plaque areas were determined with ImageJ software. Error bars represent SEM (***p < 0.01; comparisons between treatments, Multiple *t*-tests).

Given our results demonstrating that overexpression of NF-κB-65 prevents SVA-induced apoptosis and results in lower viral yields from infected cells, we investigate the direct effect of apoptosis on SVA infection. For this, replication kinetics experiments were performed in cells treated or not with Z-VAD-FMK to directly inhibit caspase-mediated apoptosis (82). As shown in Figures 10A and B, viral yields were lower in both STu and H1299 cells when apoptosis was inhibited (Z-VAD-FMK^+^).

Next, viral yields were evaluated in the supernatant or cell fraction of cell cultures treated with Z-VAD-FMK following high MOI SVA infection. Cells were infected with SVA (MOI = 5) and treated or not with apoptosis inhibitor (Z-VAD-FMK). While no differences in viral yields were detected in the cell fraction at 8 h p.i. (Figure 10C), significantly lower viral titers were detected in the supernatant of cells treated with Z-VAD-FMK when compared to control untreated cells. Additionally, the effect of apoptosis on SVA spread was assessed by plaque assays. As shown in Figure 10D, inhibition of apoptosis by Z-VAD-FMK decreased the ability of SVA to spread from cell-to-cell, as evidenced by markedly smaller viral plaques in cells treated with the apoptosis inhibitor (*p* < 0.001). Together these results suggest that induction of apoptosis late in SVA infection may be important for SVA release and/or spread from infected cells.

## Discussion

Programmed cell death or apoptosis is induced by infection with several picornaviruses (54, 55, 71, 83–85). Here, we demonstrated the occurrence of apoptosis at late times post-SVA infection and provided evidence that this process may contribute to virus release from infected cells. Notably, early in SVA infection, the apoptotic pathway is not activated, as evidenced by low Casp-3/-7 activity or lack of PARP cleavage in SVA infected cells (Figures 2A, 6B). Suppression of apoptosis early in infection likely allows the virus to complete the replication cycle before cell death/lysis (86). Inhibition of apoptosis in early stages of infection has also been described for Coxsackievirus (87) and Enterovirus 71 (88). Similar to our results with SVA, poliovirus, the prototype of the *Picornaviridae* family (89), was shown to suppress host cell death at early stages of infection and to trigger apoptosis at late times p.i. (51, 52, 90). Induction of apoptosis late in infection is thought contribute to picornavirus release from infected cells (91). Results here showing reduced SVA titers in the supernatant of cells treated with apoptosis inhibitor Z-VAD-FMK are aligned with this hypothesis.

Given that cleavage of NF-κB-p65 was detected in SVA infected cells, we investigated the potential role of SVA viral protease 3C^pro^ on this function. The 3C^pro^ is the only protease encoded by SVA and it plays a critical role during virus replication by processing the polyprotein into mature proteins (1, 92). Additionally, 3C^pro^ is important in virus-host interactions and functions as a key modulator of viral immune evasion (8, 11, 14, 16, 21, 27, 91). When SVA 3C^pro^ was transiently expressed in cell culture *in vitro*, a cleaved fragment of NF-κB-p65 similar to the one observed in the SVA infected cells, was detected. SVA 3C^pro^ also inhibited TNF-α-induced NF-κB-mediated luciferase expression, suggesting that cleavage of NF-κB-p65 leads to a decreased trans-activating activity of this transcription factor. This was also observed in the context of SVA infection, with lower NF-κB-mediated luciferase activity detected at 8–12 h post-SVA infection. An important observation to note is the fact that cleavage of NF-κB-p65 is not complete in SVA infected cells. This is reflected in the luciferase activity (Figure 5D), which shows repression of NF-κB transcriptional activity, but not complete inhibition of the transcription factor. These observations would likely explain the detection of mRNA of NF-κB-target genes late in SVA infection.

Additionally, transient expression of SVA 3C^pro^ led to cell membrane blebbing (data not shown) and cleavage of PARP, a key marker of cell death, suggesting that this viral protease is involved on host cell apoptosis (Figure 6A). This was also observed in the context of SVA infection, in which expression of SVA 3C^pro^ correlated with decreased levels of PARP and cleavage of NF-κB-p65. The ability of 3C^pro^ to induce apoptosis seems to be conserved in picornaviruses including Enterovirus 71 (93, 94), Poliovirus (95), Coxsackievirus B3 (96), and Hepatitis A virus (9).

The picornaviral 3C^pro^ contains a His-Asp-Cys catalytic triad (4, 80). This site, known as peptide-binding cleft, is located at the interface between two β-barrels (80), which is characteristic of serine proteases (97). The predicted catalytic triad of SVA 3C^pro^ consists of histidine 47 (H47), aspartate 83 (D83), and cysteine 159 (C159). To assess the importance of the proteolytic function of SVA 3C^pro^ to its pro-apoptotic activity and inhibitory effect on the NF-κB pathway, we generated two 3C mutants containing single aa substitutions at H47 (H47D) and C159 (C159R) to disrupt the catalytic triad of the protein. Notably, while expression of wild type 3C^pro^ resulted in activation of caspases and cleavage of NF-κB-p65, mutations on the catalytic triad of 3C^pro^ (H47D and C159R) abolished these phenotypes, indicating that the protease activity of SVA 3C^pro^ is essential for its function on these pathways.

Cleavage of NF-κB-p65 has also been observed during infection with poliovirus (8) and FMDV (18), and this phenotype was attributed to the expression of 3C^pro^ or L^pro^ by these viruses, respectively. Initial results here, suggested that SVA 3C^pro^ could also be potentially be involved with this function. However, since cellular caspases are also able to cleave NF-κB-p65 (64, 65), we devised experiments to dissect the mechanism underlying SVA-induced cleavage of this important transcription factor. For this, NF-κB-p65-expressing plasmids containing single or double mutations in predicted cleavage sites for the SVA 3C^pro^ and/or effector caspases were generated by site directed mutagenesis (Figure 8A). Notably, results from co-transfection and caspase inhibition experiments suggest that cleavage of NF-κB-p65 occurs due to the activity of cellular caspases and not due to the direct action of SVA 3C^pro^ (Figure 8E). Our results show that NF-κB-p65 is cleaved at a Casp-3/-6 predicted cleavage site (444LQFDTDED) when host cell apoptotic pathways are activated by expression of SVA 3C^pro^ (Figures 8B–D). Although, previous studies with poliovirus suggest that cleavage of NF-κB-p65 is mediated by 3C^pro^ (8), our findings using mutant NF-κB-p65 expressing plasmids provide evidence that, for SVA, cleavage of this important pro-survival molecule is likely secondary to caspase activation.

Several studies demonstrated that caspase-mediated cleavage of NF-κB-p65 abrogates cell survival signaling, leading to host cell apoptosis (64, 65). Notably, infection with Human Immunodeficiency virus (HIV) and African Swine fever virus (ASFV) were shown to induce caspase-mediated cleavage of NF-κB-p65 resulting in enhanced viral replication (98), or induction of apoptosis after completion of the virus replication cycle (99), respectively. Here we demonstrated the relevance of caspase-mediated cleavage of NF-κB-p65 for SVA infection. Overexpression of NF-κB-p65 suppressed SVA-induced apoptosis (Figure 9A), and resulted in significantly lower viral yields from infected cells (Figures 9B,C). These results suggest that when high levels of NF-κB-p65 are present, caspase-mediated cleavage of NF-κB-p65 may be saturated resulting in decreased levels of viral-induced apoptosis. These findings highlight the relevance of late apoptosis for SVA infection. This was confirmed with experiments in which apoptosis was inhibited throughout the virus infection cycle using the pan-caspase inhibitor Z-VAD-FMK. Results from these experiments demonstrated lower viral yields from cells in which apoptosis was suppressed. These findings suggest that SVA induces apoptosis late in infection, presumably as a mechanism to facilitate virus release and/or spread from infected cells (Figure 10C). Indeed, the primary mode of spreading of non-enveloped viruses is cell lysis (100), and apoptosis could allow viruses to use cell remnants for viral transmission, while avoiding the host immune response (58).

In summary, results here show that SVA modulates important host cell pathways, including NF-κB and apoptosis, throughout the infection cycle. The proteolytic activity of SVA 3C^pro^ is essential to induction of apoptosis, however the precise function of the viral protease to this process remains unknown. Our data demonstrate that induction of apoptosis late in infection is a critical process for SVA infection *in vitro*. Results showing induction of apoptosis *in vivo* suggest that this event may also have direct implications on SVA infection and pathogenesis in the natural swine host.

## Bioethics

All animal studies were carried out in accordance with the principles of the the Animal Welfare Act, the Public Health Service (PHS) Policy on Humane Care and Use of Laboratory Animals, and recommendations of the Guide for the Care and Use of Laboratory Animals. The protocols were approved by the SDSU IACUC (15-095A and 16-002A).

## Data Availability

All datasets generated for this study are included in the manuscript and/or the supplementary files.

## Author Contributions

MF designed, performed, and analyzed the experiments and data. MM, JO, LJ, and SL developed reagents and helped with experiments. DD planned experiments, analyzed data, and secured funding. MF and DD wrote the manuscript.

### Conflict of Interest Statement

The authors declare that the research was conducted in the absence of any commercial or financial relationships that could be construed as a potential conflict of interest.
